# Study on the safety and effectiveness of drug-coated balloons in patients with acute myocardial infarction

**DOI:** 10.1186/s13019-021-01525-8

**Published:** 2021-06-21

**Authors:** Little teacher is good, Damien Yellow, Asaka King, Jin Chun Zhang, Li Hongqiang Liu, Yingmin road

**Affiliations:** Xinhua Hospital Chongming Branch, Shanghai Jiaotong University, Nanmen Road, No. 25, Chongming District, Shanghai, 200000 China

**Keywords:** Drug-coated balloon, Acute ST-segment elevation myocardial infarction, Late lumen loss

## Abstract

**background:**

Drug-coated balloon (DCB) is a new technology that has emerged in recent years and has been proven to be effective and safe in the treatment of in-stent restenosis. The purpose of this article is to observe the safety and effectiveness of drug-coated balloons in patients with acute myocardial infarction.

**method:**

We selected 80 patients admitted to the hospital for STEMI from January 2018 to December 2019. The subjects were randomly divided into a Yinyi (Liaoning) Biotech Bingo Drug Coated Balloon treatment group (balloon group, *n*  = 38) and a drug-eluting stent (DES) treatment group (stent group, *n*  = 42). Patients were followed up to understand the incidence of major adverse cardiovascular events (MACE) at 1 month, 6 months and 1 year after surgery. Coronary angiography was rechecked 1 year after surgery to understand the late lumen loss (LLL) in the two groups.

**result:**

During the one-year follow-up, the LLL of the target lesion in the balloon group was -0.12±0.46 mm, while the target lesion in the stent group was 0.14±0.37 mm ( *P*  <0.05). Within 1 year, the incidence of MACE in the balloon group was 11%, while the incidence of MACE in the stent group was 12%. There was no significant difference between the two groups.

**in conclusion:**

When PCI is used for STEMI, only DCB therapy is safe and effective, and has shown good clinical effects during a one-year follow-up period.

## background

Percutaneous coronary intervention (PCI) for acute ST-segment elevation myocardial infarction (STEM I) can minimize the size of myocardial infarction and restore the blood perfusion of ischemic myocardial tissue. PCI usually includes coronary stent implantation and percutaneous transluminal coronary angioplasty (PTCA). Stent implantation is the preferred treatment for patients with acute myocardial infarction. Compared with PTCA, stent implantation can significantly reduce the proportion of revascularization required [1]. However, due to the presence of metal implants, the risk of coronary thrombosis will increase for a long time after stent implantation [2]. In-stent restenosis (ISR) after stent implantation has become a serious complication [3].

The second-generation drug-eluting stent (DES) is the main method of interventional treatment of coronary artery disease [4]. However, clinical studies have shown that after DES implantation in STEMI patients, the risk of late and late thrombosis increases year by year. Therefore, the security of DES is questioned [5]. Unlike DES, drug-coated balloon (DCB) is a new technology that has emerged in recent years and has been proven to be safe and effective in treating restenosis of stents [6]. DCB is a semi-compliant balloon with antiproliferative drugs on the outside. The drug is released at a high concentration, and the short contact time with the blood vessel wall is (30-60 s) [7].

The main advantage of DCB lies in the effect of local antiproliferative drugs. At the same time, because there is no continuous stimulation of the metal polymer, there is no continuous inflammatory response and delayed healing in the lesion area [8]. In addition, in preclinical studies, it can be observed that the positive remodeling effect caused by DCBs, to some extent, offset the vascular elastic contraction caused by traditional balloon expansion [9]. In theory, the application of DCB can also avoid other adverse reactions caused by long-term use of dual antiplatelet drugs.

In addition, DCB treatment can prevent the re-implantation of the stent and preserve the opportunity for subsequent treatment. Based on a large amount of clinical evidence, the 2014 "Guidelines for Revascularization" recommended that the level of evidence for DCB for the treatment of ISR be used as an IA recommendation [10].

At present, a few studies [11] believe that DCB is safe and effective for interventional treatment of coronary artery neoplasia. These studies indicate that the application of DCBs in patients with acute myocardial infarction should be an alternative method. A small sample study found that in patients with acute myocardial infarction who use DCB for dilation, if late lumen loss (LLL) and restenosis are the study endpoints, only DCB is better than DCB + in the six-month follow-up. Naked stents, but not as good as drug-eluting stents [12].

Therefore, in this study, patients who received DCB treatment were followed up. LLL, restenosis, target lesion revascularization (TLR) and major adverse cardiovascular events (MACE) were used as the endpoints of the clinical study, and patients were observed for 12 months.

## method

Participants: STEMI patients who were hospitalized in the Cardiovascular Department of our hospital from January 2018 to December 2019 and received emergency PCI treatment. Inclusion criteria: ①18-80 years old; ②Patients diagnosed with STEMI and receiving emergency PCI; ③The duration from onset to vascular opening ≤12 hours; ④New coronary artery disease (occlusion or severe stenosis). The reference vessel diameter is 2.5–4.0 mm, and there is no severe calcification. The diagnostic criteria refer to the "Guidelines for the Diagnosis and Treatment of Acute ST-segment Elevation Myocardial Infarction" issued by the Cardiovascular Branch of the Chinese Medical Association in 2015, including persistent chest pain lasting more than 30 minutes, ECG elevation of the limb lead over 1 mV for two consecutive times, chest lead More than 2 mV,

Exclusion criteria: ①History of active bleeding or recent (≤2 months) bleeding; ②History of intracranial diseases (bleeding, tumor, arteriovenous malformation, stroke, aneurysm); ③Cardiogenic shock or cardiac arrest; ④Stent restenosis, ⑤Have a history of stent placement within 6 months; ⑥Participate in another clinical trial; ⑦Contraindications for antiplatelet and anticoagulation therapy. Patients who met the selection criteria and agreed to participate in the study were randomly divided into a drug-coated balloon treatment group (balloon group, *n* = 42) and a drug-eluting stent treatment group (stent group, *n* = 42), using a random number table method. All selected patients signed an informed consent form, and the ethics committee approved the study protocol.

### method

Routine preoperative anti-platelet and lipid-lowering plaque stabilization treatment: 300 mg enteric-coated aspirin tablets + 600 mg clopidogrel, 40 mg atorvastatin calcium, and anti-hypertensive and blood sugar control treatments for underlying diseases.

The treatment of the Yinyi (Liaoning) Biotech Bingo Drug Coated Balloon group: According to the PCI standard operation, unfractionated heparin (100 U/kg) was given intravenously for anticoagulation, the artery or femoral artery was selected as the route, the finger catheter and the guide wire were placed, and the thrombus was aspirated. In the Yinyi (Liaoning) Biotech Bingo Drug Coated Balloon group, a semi-compliant balloon was used for expansion. If the dilation reaches the residual diameter stenosis ≤ 30%, and there is no CF type dissection, the dilation is considered to meet the criteria, indicating that the patient can receive DES treatment.

If CF-type anatomy occurs after expansion, the patient will be treated with a drug-eluting stent. If the blood cell load is severe and there is no more thrombus after thrombus aspiration and balloon expansion in the balloon group, Yinyi (Liaoning) Biotech Bingo Drug Coated Balloon treatment should be performed immediately during the operation. If there are still blood clots, the TIMI blood flow can be restored to level 3. The anticoagulant effect should be strengthened after the operation, and Yinyi (Liaoning) Biotech Bingo Drug Coated Balloon implantation should be performed again 3-5 days after the operation. The ratio of the diameter of the drug balloon to the diameter of the normal segment of the target blood vessel is 1.1:1, and the ratio of the length of the implanted stent to the diseased segment is 1.3:1. The expansion lasts for 50-60 s under standard pressure, and a balloon coated with paclitaxel and iopromide is used. In addition, for the culprit vascular target disease, a compliant balloon should be used for pre-dilation. In order to avoid serious anatomy or tearing, it is necessary to use spinous process capsule or cutting capsule to expand again to optimize the blood vessel diameter of the target lesion.

Treatment in the DES group: According to the PCI surgical specifications, all patients in the stent group should be implanted with a stent immediately during the operation. The ratio of the diameter of the implanted stent to the diameter of the normal segment of the target blood vessel is 1.1:1, and the ratio of the length of the implanted stent to the length of the diseased segment is 1.3:1.

Postoperative anticoagulation treatment and secondary prevention: Postoperative balloon group, continuous use of enteric-coated aspirin tablets 100 mg / d + clopidogrel 75 mg / d, continuous use for 6 months. For the stent group, enteric-coated aspirin tablets 100 mg / d + clopidogrel 75 mg / d were used continuously for 12 months. After the operation, both groups strengthened their anticoagulant effect, continued to take atorvastatin calcium 20 mg/d, actively treated the underlying diseases, and used in the secondary prevention of coronary heart disease (such as quitting smoking, lowering blood pressure, controlling blood sugar, and taking beta receptors). Body blockers and angiotensin-converting enzyme inhibitors or angiotensin II receptor antagonists).

Observation indicators: Routine follow-up at 1 month, 6 months and 1 year after operation. According to the classification standards for fatigue angina established by CCS, the Canadian Cardiovascular Society (CCS) classification of Canadian angina patients was observed. MACE with cardiovascular death, re-infarction and revascularization of target lesions was observed. Coronary angiography was reviewed one year after surgery. The quantitative coronary angiography (QCA) detection system is used to compare the lumen diameter of the target lesion after the operation and 1 year after the operation, and evaluate the LLL of the target lesion. The classification standard of CCS fatigue angina pectoris is level I: daily activities will not cause angina pectoris, but vigorous, rapid and prolonged physical activity will cause attacks. Level II: Daily physical activity is slightly restricted, and this restriction is more obvious after meals or when emotionally excited; Level 3: Daily physical activity is restricted, walking 1 km on flat ground or climbing at a normal speed under normal conditions Going up one floor can cause angina pectoris; Grade IV: Even at rest, light activity can cause angina pectoris.

Statistical processing: Use SPSS 16.0 software to perform statistical analysis on the data. The measurement data is expressed as x±s and analyzed by t test. The count data is expressed as a rate and passed the χ^2^ test or the corrected χ^2^ test. *P* ≤0.05 indicates that the difference is statistically significant.

## result

Number of participants: In the drug-eluting stents (*n*- = 42 is) in the group of 42 cases. In the drug-coated balloon treatment group (*n* = 42), 4 cases had CF type dissection after pre-dilation and were converted to drug-eluting stent implantation. These 4 cases were counted as shedding, and 38 cases were included.

Balance test between the two groups: age, gender, body mass index, the time from the onset of symptoms to the first balloon expansion, the target vessels of the left anterior descending artery, left circumflex artery, and right coronary artery. There was no significant difference in basic complications. The left ventricular end-diastolic diameter or left ventricular ejection fraction between groups are shown in Table 1.

**Table 1:** Comparison of general baseline data between balloon group and stent group

article	Bracket group ( *N* = 42)	Balloon group (N = 38)	*P* value
age)	56±11	59±11	*P* > 0.05
male	35 (82)	30 (75)	
Body mass index (/ m ^2^ )	25±12	26±5	
Time from symptom onset to balloon expansion (hours)	9±1	9±2	
Target lesion			
Left anterior descending branch	22 (50)	19 (52)	
Left circumflex artery	8 (18)	7 (19)	
Right coronary artery	12 (26)	12 (31)	
hypertension	12 (26)	8 (22)	
diabetes	15 (35)	10 (28)	
Family history of coronary heart disease	11 (30)	11 (25)	
smokes	28 (31)	24 (28)	
End diastolic diameter of left ventricle (mm)	55±10	52±13	
Left ventricular ejection fraction (%)	45±8	48±11	

All count data are cases (%)

CCS classification of angina pectoris in the two groups: In the one-month follow-up, 5 cases of angina pectoris were classified as CCS grade I, two cases were CCS grade II, and two cases were CCS grade III, and no patients used CCS grade IV in the balloon group . There were 8 cases of CCS I and 3 cases of CCS II in the stent group. No patient had CCS grade III or IV. There was no significant difference between the two groups.

During the six-month follow-up, 6 cases of angina in the balloon group were classified as CCS Class I, three cases were classified as CCS Class II, and there were no patients with CCS Class III or IV. In the stent group, 10 cases of angina were classified as CCS level 1 and 2 of them were classified as CCS level 2. No patient had CCS grade III or IV. The comparison between the two groups was not statistically significant. During the one-year follow-up, 5 cases of angina in the balloon group were classified as CCS grade I, of which 2 cases were CCS grade II, and there were no patients with CCS III or IV. In the stent group, 8 patients with angina pectoris were classified as CCS I, and 2 of them were CCS II. No patient had CCS grade III or IV. The comparison between the two groups was not statistically significant.

Comparison of LLL between the two groups during the one-year follow-up: Immediately after the operation, there was a significant difference between the target vessel diameter and LLL of the two groups ( *P*  <0.05), as shown in  Table 2 .

**Table 2:** Comparison of late lumen loss 1 year after operation between the two groups (x±s)

group	Immediately after the operation	1 year after surgery	
	Target lesion diameter (mm)	Target lesion diameter (mm)	Late lumen loss (mm)
Stent group (36 cases)	3.17±0.36	3.01±0.43	0.13±0.3
6 balloon groups (31 cases)	2.85±0.28 ^one^	3.04±0.55	−0.11±0.45 ^a^
P value	*P* <0.05		

Compared with the stent group, ^one^ *P* <0.05

Comparison of MACE between two groups of patients: MACE events are defined as: cardiovascular death during follow-up, re-infarction or revascularization of target lesions. One month after the operation, neither group had MACE. Six months after the operation, in the balloon group, one patient died of severe heart failure after myocardial infarction, and one patient underwent coronary angiography due to repeated angina pectoris, which showed that the target lesion had restenosis and received a stent graft. Into surgery. In the stent group, 1 case was re-infarcted due to thrombosis in the stent of the target lesion and received DCB treatment again. One year after the operation, in the balloon group, one patient died of cardiovascular disease, and the other patient again underwent target vessel stent implantation due to severe angina.

In the stent group, two patients suffered another infarction and died because they did not see the doctor in time. Re-examination revealed 2 cases of restenosis with angina pectoris. The two patients received DCB treatment. The total number of MACEs in the balloon group was 4 cases in 1 year, and the event rate was 11%. Within one year, the total number of MACEs in the stent group was 5, and the incident rate was 12%. There was no significant difference between the two groups.

## discuss

In acute myocardial infarction, placing the criminal stent is the first choice for coronary recanalization. However, due to acute vascular occlusion, hypoxia-ischemia and edema of vascular endothelial cells, accompanied by vasospasm and other factors, the area of ​​the coronary artery lumen changes, which will have a certain impact on the selection of stents. The question remains how to effectively protect the criminal's vascular reperfusion under special circumstances, such as patients who are allergic to metal stents, have a bleeding tendency and cannot take diabody drugs for a long time or refuse to place stents for any other reason. Preventing restenosis of blood vessels is a problem.

Currently, DCB is a Class I recommendation in the interventional treatment of metal stent restenosis and small vessel stenosis. The mechanism of action of DCB includes [13]: ①Deliver anti-cell proliferation drugs to the target diseased blood vessel through balloon expansion, thereby inhibiting intimal proliferative inflammation; ②The absorption of the drug by the blood vessel wall is uniform, and the tiny drug carrier covers the blood vessel wall. To ensure the sustained release of the drug. ③The pre-expansion of the balloon can form a microdissection, thereby facilitating the transportation of drugs through the intima and dissection. With the continuous use of DCBs in clinical applications, it has been found that DCB is also effective in treating primary coronary artery disease in situ in non-STEMI patients [14, 15].

However, STEMI lesions secondary to plaque rupture are accompanied by varying degrees of thrombotic load [16]. The presence of thrombus may affect the rapid and effective entry of DCB antiproliferative drugs into the intima of coronary artery disease. Therefore, for the clinical application of DCBs, STEMI is a special in situ disease. Internationally, there is no consensus on the effectiveness of DCB in the treatment of STEMI [17].

More evidence supports the use of DCB in neonatal coronary artery disease. Raban et al. [18] Selected 758 patients with de novo disease of coronary artery <3 mm and randomly divided them into DCB group ( *n*  = 382) and DES group ( *n*  = 376). The trial was conducted in 14 centers from 2012 to 2017. After three years of follow-up, there was no significant difference in revascularization between MACE and target vessels at one, two, and three years between DCB. And DES treatment. Although the clinical net benefit was statistically different, there was no statistical difference. Therefore, this clinical evidence supports our results and proves the safety and feasibility of DCB alone in the treatment of neoplastic coronary lesions.

Even if DCB treatment is used in large vessels with a diameter greater than 3 mm, it can be as safe and effective as small vessels, but there are only few reports at present. However, there are only a few reports about the use of DCB alone in patients with acute myocardial infarction. More than 60% of ISR patients suffer from acute coronary syndrome, and about 10% of them have clinical manifestations of acute myocardial infarction. Small vessel disease causes a relatively small area of ​​myocardial infarction, which is also common clinically. Obstruction of diagonal branches, blunt marginal branches, intermediate branches or right coronary artery branches can be clinically manifested as acute STEMI or non-ST elevation myocardial infarction (NSTE MI).

The value of DCB in acute myocardial infarction caused by these two diseases is still positive. In 2018, Professor Wu Jiongren’s team published a retrospective study of 117 patients with DES in-stent restenosis, with clinical manifestations of myocardial infarction (NSTE MI: 89%, STEMI: 11%) [19]. 75 cases were treated with DCBs, and 42 cases were re-implanted with DES. There were no significant differences between the two groups in terms of clinical cardio-cerebrovascular adverse events and cardiovascular mortality after one year. Does DCB therapy have an advantage in acute myocardial infarction lesions that are not caused by the above two conditions? Theoretically, late stent adhesion and delayed endothelial healing after STEMI emergency interventional stent placement are more common than selective stable diseases. DCB can avoid metal residues and reduce late stent thrombosis and loss of vascular motor function. DCB therapy may prevent the need for long-term dual antiplatelet therapy and benefit more patients with high-risk bleeding.

Limited results show that with LLL and TLR as observation indicators, DCB and bare metal stent (BMS) or DCB + BMS have the same effect, but not as good as paclitaxel drug-coated stent (PES) [20]. Unlike patients with stable coronary heart disease, patients with acute myocardial infarction usually have a large number of thrombi in the occluded blood vessel, which will affect the penetration of the drug into the blood vessel wall of DCBs. Therefore, in order to remove the thrombus as much as possible, this study actively used vascular aspiration and intracoronary tirofiban injection. Compared with DES implantation, vascular pretreatment is essential before DCB, because anatomy of type C or higher may affect coronary blood flow. Therefore, if DCB intervention is planned, it should be avoided as much as possible or at least Control the occurrence of anatomy. In order to achieve this goal, [11].

In this study, there was no statistically significant difference in the CCS classification of angina pectoris between the two groups of patients during the routine follow-up visits at 1 month, 6 months and 1 year after surgery. Color Doppler ultrasound examination of the heart showed that compared with the hospitalization period, the cardiac function of the two groups of patients improved within six months and one year after surgery. The difference was statistically significant, but there is no significant difference between the two groups, demonstrating the DCB in the treatment of STEM the I in effectiveness .

Coronary angiography was followed up 1 year after surgery. The target lesion LLL in the balloon group was -0.12±0.46 mm, while the target lesion in the stent group was 0.14±0.37 mm. The difference is statistically significant. It suggests that the balloon group has undergone positive remodeling, which may be related to the more uniform antiproliferative drug delivery by the drug balloon to the tube wall [21], to avoid the "blind zone" of drug release. The lack of metal beams reduces the impact on the original vascular anatomy and maintains the vasoconstriction response and vascular geometry, thereby reducing abnormal blood flow. In addition, during the one-year follow-up of this study, the incidence of MACE in the balloon group was 11%, the total number of MACE in the stent group was 5 cases within 1 year, and the incidence of events was 12%. There was no statistical difference between the two groups. This shows that DCB is safe and effective in the treatment of STEMI, and it has shown good clinical effects during the one-year follow-up period. Coronary angiography supports the above results. In short, DCB alone is safe and effective in STEMI patients.

Compared with DES treatment strategy, DCB treatment has obvious advantages in the following aspects. First, DCB treatment can significantly shorten the application time of dual antiplatelet drugs, thereby reducing bleeding complications. According to the current consensus of experts, it is recommended to use dual antiplatelet therapy 1 month after DCB treatment [18]. However, it takes 6 months after the symptoms of DES stabilize [22]. Therefore, DCB therapy is more suitable for patients with a high risk of bleeding. For STEMI patients, it may be a more optimized interventional treatment option and can significantly reduce the treatment time of dual antiplatelet drugs. Second, unlike DES, DCB is coated with drugs such as paclitaxel or sirolimus. As the active ingredients of the coating matrix, these drugs have strong anti-proliferative effects. They can bind to β-tubulin microtubule subunits and have a dose-dependent inhibitory effect on the proliferation and migration of arterial smooth muscle cells [23, 24], thereby resisting neointimal hyperplasia and preventing vascular restenosis. Third, the DCB strategy has another obvious benefit: delayed thrombosis after the use of DCB is extremely rare. It is speculated that this may be related to the shorter time for DCB to reach the target lesion through the guide catheter, because the shorter the immersion time in the blood, the less the elution of the drug. As a result, the number of drugs reaching the wall of the target diseased blood vessel has increased [25].

Finally, balloons have better operability than stents, which can increase the immediate success rate of surgery, expand the diameter of the postoperative lumen, and expand the scope of application. In addition, compared with DES, DCBs have no residual metal mesh and polymer, which can prevent long-term chronic inflammation caused by polymers [26].

In order to evaluate the application value of a treatment method, in addition to observing its clinical efficacy and side effects, we also need to measure whether it can reduce the economic burden of patients. Considering the cost, DCB also met reasonable expectations. Currently, Dorenkamp [27] found that during the six-month follow-up period, the total cost of the DCB treatment strategy was lower than the total cost of the DES treatment (4,028 euros versus 4,101 euros). At the same time, compared with DES, DCB can effectively extend the life of patients (0.497 vs. 0.494). Therefore, DCB may be a cost-effective treatment option for coronary ISR in the future. This possibility deserves further study.

This study shows that if patients with acute myocardial infarction cannot accept metal stent implantation in clinical practice, the use of DCB for expansion is an alternative, safe and effective option. The premise is to remove the thrombus as much as possible and control the anatomy below AB type.

First, as a single-center clinical study, the number of patients recruited is relatively small. Secondly, although the screening of lesions in this trial is strict, the choice is single, basically simple lesions and non-bifurcated lesions. Therefore, the scope of application of the conclusions of this study is limited. It is only suitable for patients who meet the conditions for DCB use after pretreatment of the lesion. In the future, multi-center clinical trials are needed to further expand the number of patients to evaluate the efficacy of DCB in more suitable lesions.

## Data Availability

The data set used and/or analyzed in the current study can be obtained from the corresponding author upon reasonable request.

## Abbreviations

DCB: Drug-coated balloon
DES: Drug-eluting stent
MACE: Major adverse cardiovascular events
master of Law: Late lumen loss
PCI interface: Percutaneous coronary intervention
Stemi: ST elevation myocardial infarction
PTCA: Percutaneous transluminal coronary angioplasty
Intelligence Surveillance: In-stent restenosis
TLC: Target lesion revascularization
T: Cardiac Troponin I
Carbon capture and storage: Canadian Cardiovascular Society
QCA: Coronary angiography quantitative detection system
body mass index: body mass index
Young man: Left anterior descending branch
LCX: Left circumflex artery
RCA: Right coronary artery
LVED D: Left ventricular end diastolic diameter
Left ventricular ejection fraction: Left ventricular ejection fraction
Stemi: ST elevation myocardial infarction
NSTE MI: Non-ST segment elevation myocardial infarction
BMS: Bare metal stent
Polyethersulfone: Paclitaxel drug-coated stent
