# Correction to: Study on the safety and effectiveness of drug-coated balloons in patients with acute myocardial infarction

**DOI:** 10.1186/s13019-021-01629-1

**Published:** 2021-09-03

**Authors:** Xiaojiao Hao, Damin Huang, Zhaoxia Wang, Jinchun Zhang, Hongqiang Liu, Yingmin Lu

**Affiliations:** Department of Cardiology, Xinhua (Chongming) Hospital, School of Medicine, Shanghai Jiaotong University, Shanghai, 200000 China

## Correction to: Journal of Cardiothoracic Surgery (2021) 16:178 https://doi.org/10.1186/s13019-021-01525-8

The original article [1] contained errors in the manifestation of the authorship which have since been corrected.
